# Supplementary material for: Reconstructed eight-century streamflow in the Tibetan Plateau reveals contrasting regional variability and strong nonstationarity
Source: Nat Commun. 2022 Oct 27;13:6416. doi: 10.1038/s41467-022-34221-9 (PMC9613640; doi:10.1038/s41467-022-34221-9)
Supplement: Supplementary file 1 — Supplementary information [file 41467_2022_34221_MOESM1_ESM.pdf]

Supplementary Information for

Reconstructed eight-century streamflow in the Tibetan Plateau reveals contrasting regional variability and strong nonstationarity

Yenan Wu[1], Di Long[1*], Upmanu Lall[2], Bridget R. Scanlon[3], Fuqiang Tian[1], Xudong Fu[1], Jianshi Zhao[1], Jianyun Zhang[4], Hao Wang[5], and Chunhong Hu[5]

1. State Key Laboratory of Hydroscience and Engineering, Department of Hydraulic Engineering, Tsinghua University, Beijing 100084, China

2. Department of Earth and Environmental Engineering, Columbia University, New York, NY 10027, USA

3. Bureau of Economic Geology, Jackson School of Geosciences, The University of Texas at Austin, Austin, TX 78758, USA

4. State Key Laboratory of Hydrology-Water Resources and Hydraulic Engineering, Nanjing Hydraulic Research Institute, Nanjing 210098, China

5. State Key Laboratory of Simulation and Regulation of Water Cycle in River Basin, China Institute of Water Resources and Hydropower Research, Beijing 100038, China

*Correspondence to Di Long (dlong@tsinghua.edu.cn)

Supplementary figures

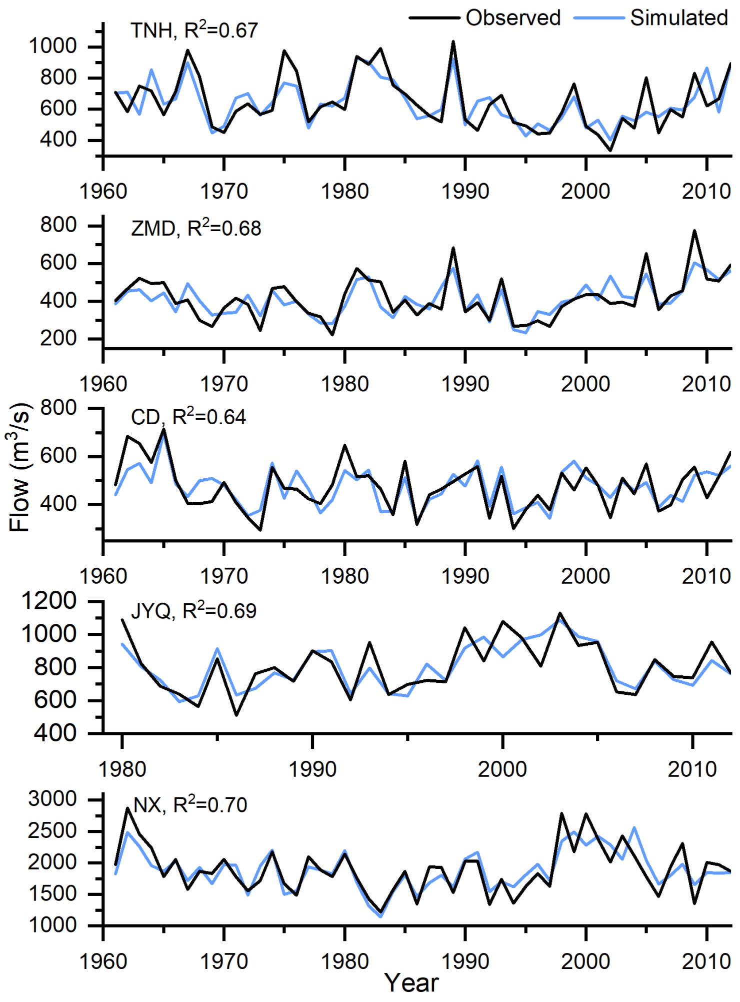

Supplementary Figure 1 Observed streamflow versus reconstructed streamflow at five streamflow gauging stations (i.e., Tangnaihai (TNH) on the Yellow River, Zhimenda (ZMD) on the Yangtze River, Changdu (CD) on the Lancang-Mekong River, Jiayuqiao (JYQ) on the Nu-Salween River, and Nuxia (NX) on the Yarlung Zangbo-Brahmaputra River) in headwater regions of major rivers in South and East Asia during 1961−2012. The reconstruction performance represented by R^2 for each reconstructed model is also given.

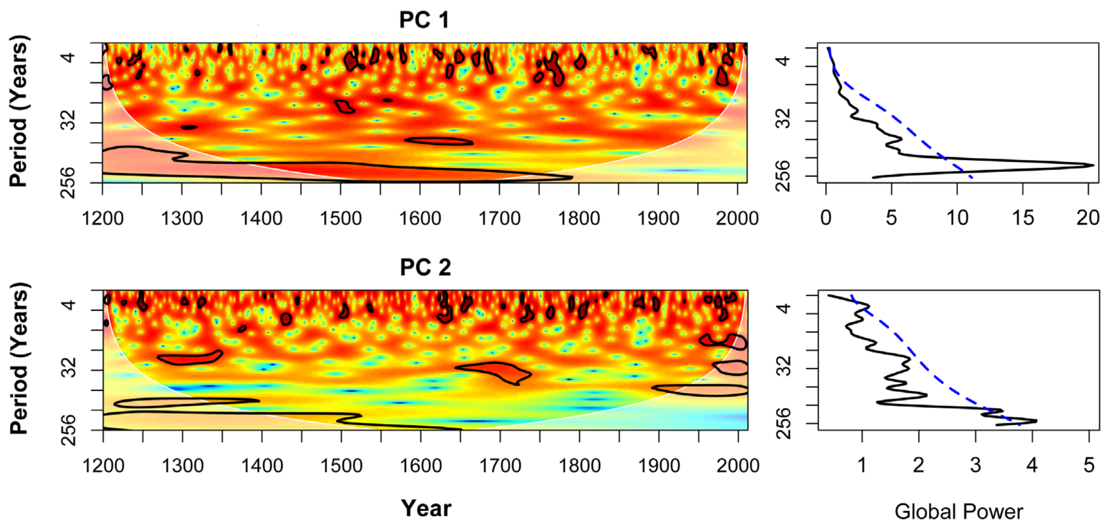

Supplementary Figure 2 Wavelet power spectra analysis for the leading PCs of reconstructed streamflow during 1200−2012. The left panel illustrates wavelet transforms and the right panel shows the global wavelet power spectrum of PC1 and PC2. Solid black contours enclosed in a temporal space are statistically significant at a 5% significance level of a white noise process. The white line shows the cone of influence where the edge effects may impact the results. Blue dotted lines in global power spectra (GWS, right panel) show a 95% confidence level of white noise. The PC1 wavelet spectra show interannual oscillations at 2–8 year scales that mainly occurred in 1500s–1800s and the post 1950s. There were also some sporadic intermittent decadal cycles and a strong multi-decadal oscillation at 120–240 year time scales during 1200s–1800s. This prolonged multi-decadal mode suggests that the persistence of dry and wet conditions during 1200s–1800s was longer than that during the instrumental period. The PC2 CWT spectra show significant multi-decadal oscillations (centered at ~80 and ~160 years) over 1200s–1600s and parts of the oscillations are located outside of the cone of influence. After the 1600s, the dominant periodicities in streamflow variability of PC2 became shorter. Variability in PC2 during the 20th century is a combination of significant interannual (2–7 years), decadal (10–32 years), and multi-decadal (~50 years) scale variability. Both PC1 and PC2 GWS results exhibit statistically significant oscillations at interannual (2–8 years) and multi-decadal (> 100 years) periodicities.

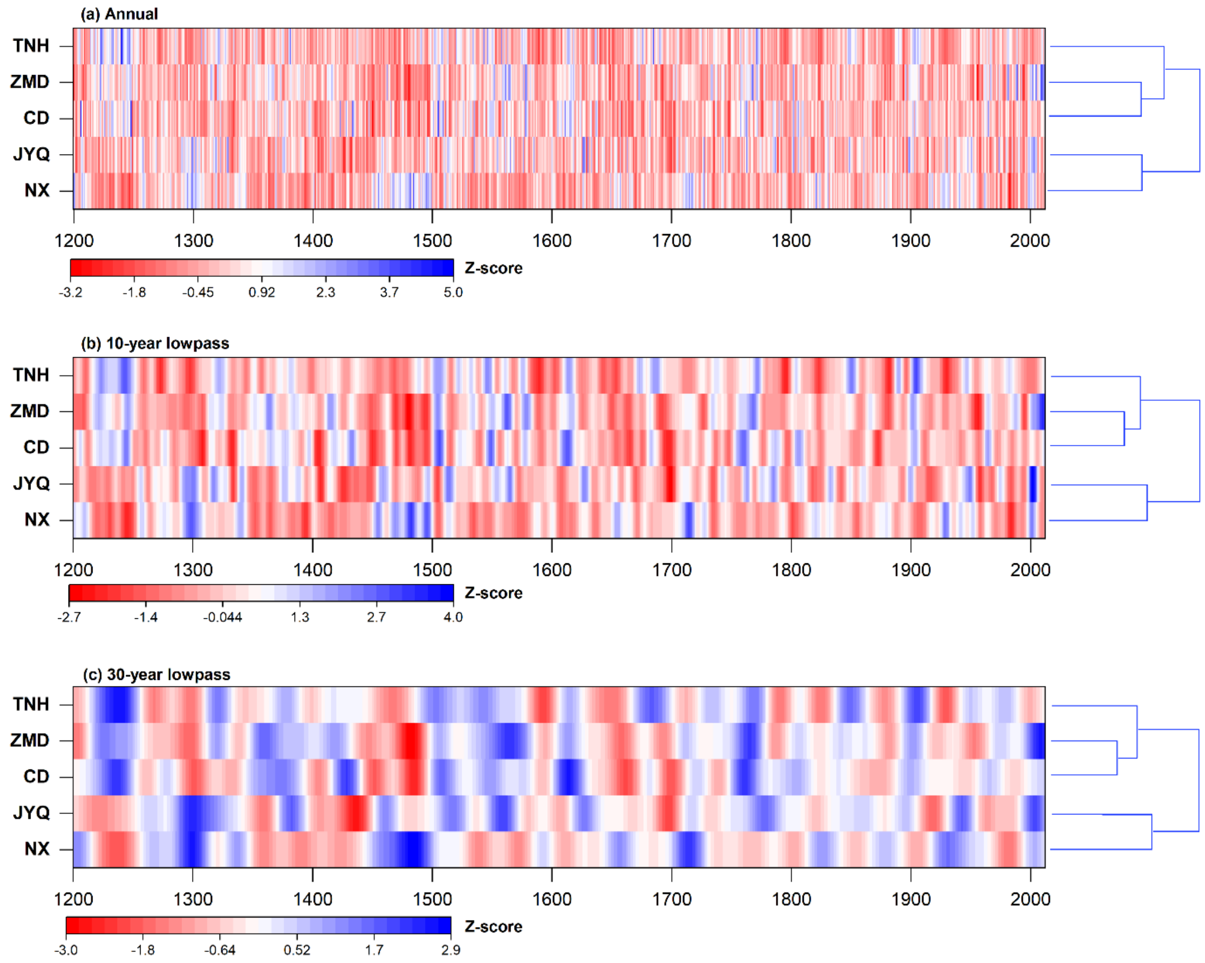

Supplementary Figure 3 Dendrograms and cluster spatializations in two clusters for the five gauging stations based on (a) the scaled annual reconstructed streamflow, (b) the scaled 10-year low pass reconstructed streamflow, and (c) the scaled 30-year low pass reconstructed streamflow during 1200–2012. These three low pass streamflow time series show the same clusters in space, including the TNH, ZMD, and CD gauging stations in the northern SETP, but the JYQ and NX gauging stations in the southern SETP.

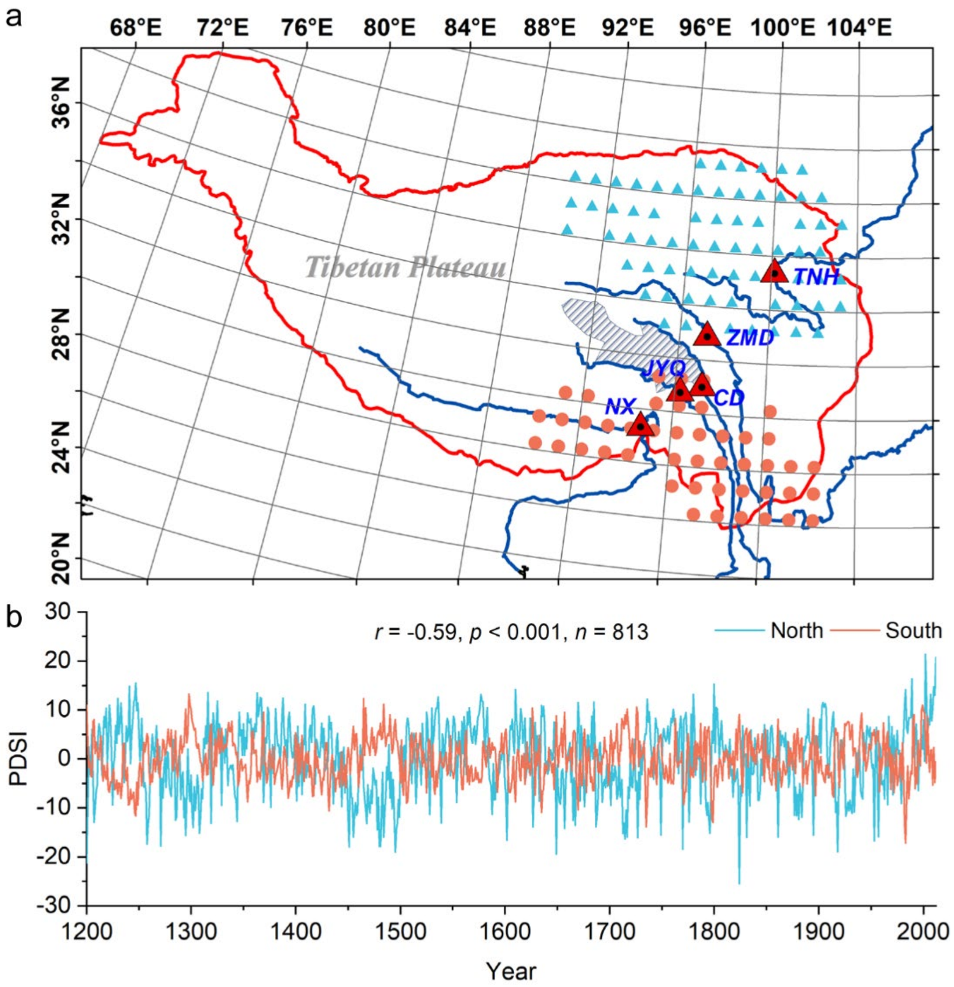

Supplementary Figure 4 Comparison of PDSI proxy data located in the northern and southern SETP during 1200−2012. The first principal component ($PC1_{North}$, 72% of variance explained, blue line in Fig. S4b) of 71 PDSI grid cells in the northern region (blue triangles in Fig. S4a) and the first principal component ($PC1_{South}$, 45% of variance explained, red line in Fig. S4b) of 45 PDSI grid cells in the southern region (red circles in Fig. S4a) show significant negative correlation (r = -0.59, p < 0.01, n = 813) during 1200−2012. Shaded areas in Fig. S4a represent the Tanggula Mountains from northwest to southeast.

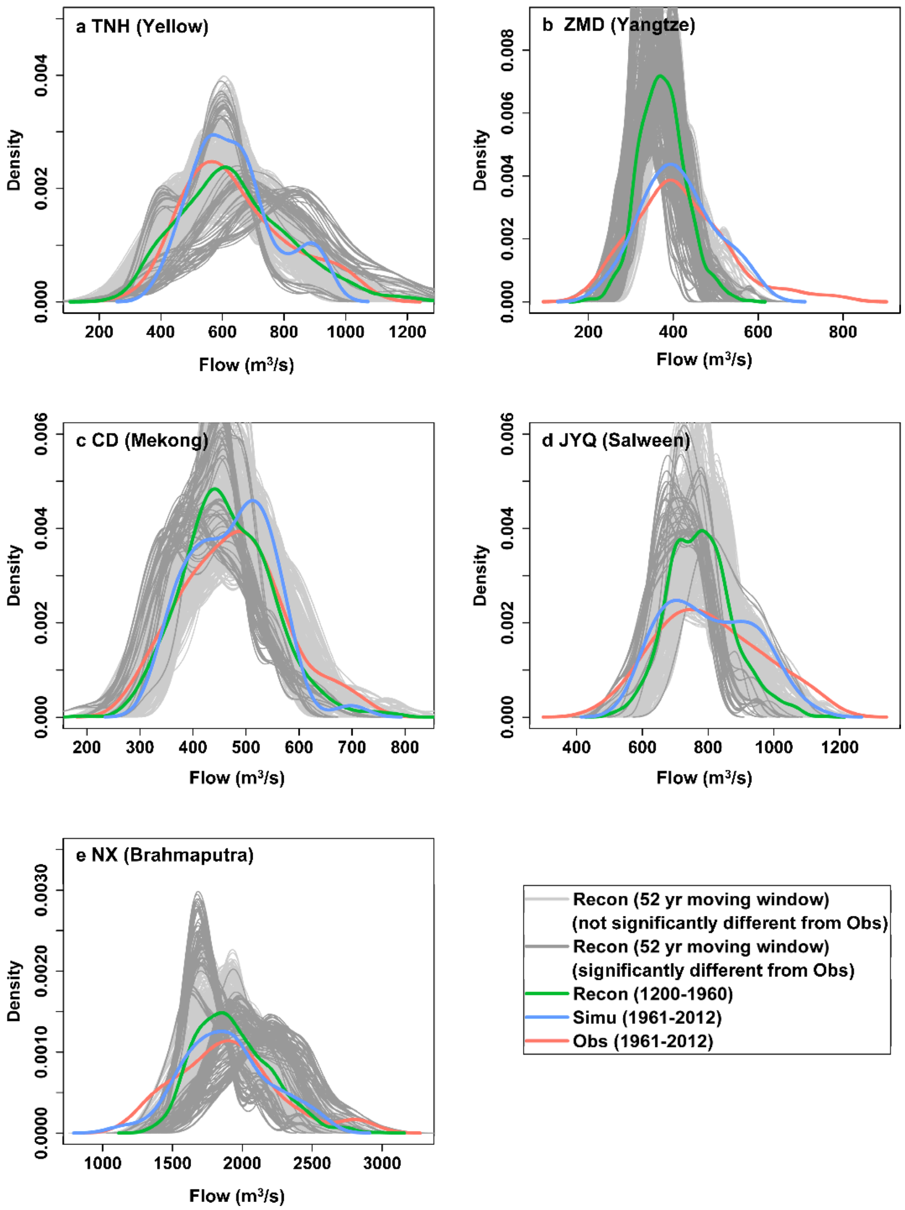

Supplementary Figure 5 Kernel density plots of reconstructed streamflow (green lines) during 1200−1960 (1200−1979 for JYQ), simulated (blue lines), and observed (red lines) streamflow during 1961−2012 (1980−2012 for JYQ). Light grey lines represent the kernel density of reconstructed streamflow within each 52-year moving window (33-year moving window for JYQ) that is not significantly different from the kernel density of the observed data based on the Kolmogorov-Smirnov test. Dark grey lines are the same as the light grey lines but for the density of reconstructed streamflow that is from different probability distributions ($p<0.05$) with the observed data. The K-S test was also used to test if the probability distribution differs between the long-term reconstructions (1200−1960) and observations. The probability distribution of long-term reconstructed streamflow (1200–1960) at ZMD (Yangtze) also significantly differs ($p<0.05$) from the probability distribution of instrumental records (red line).

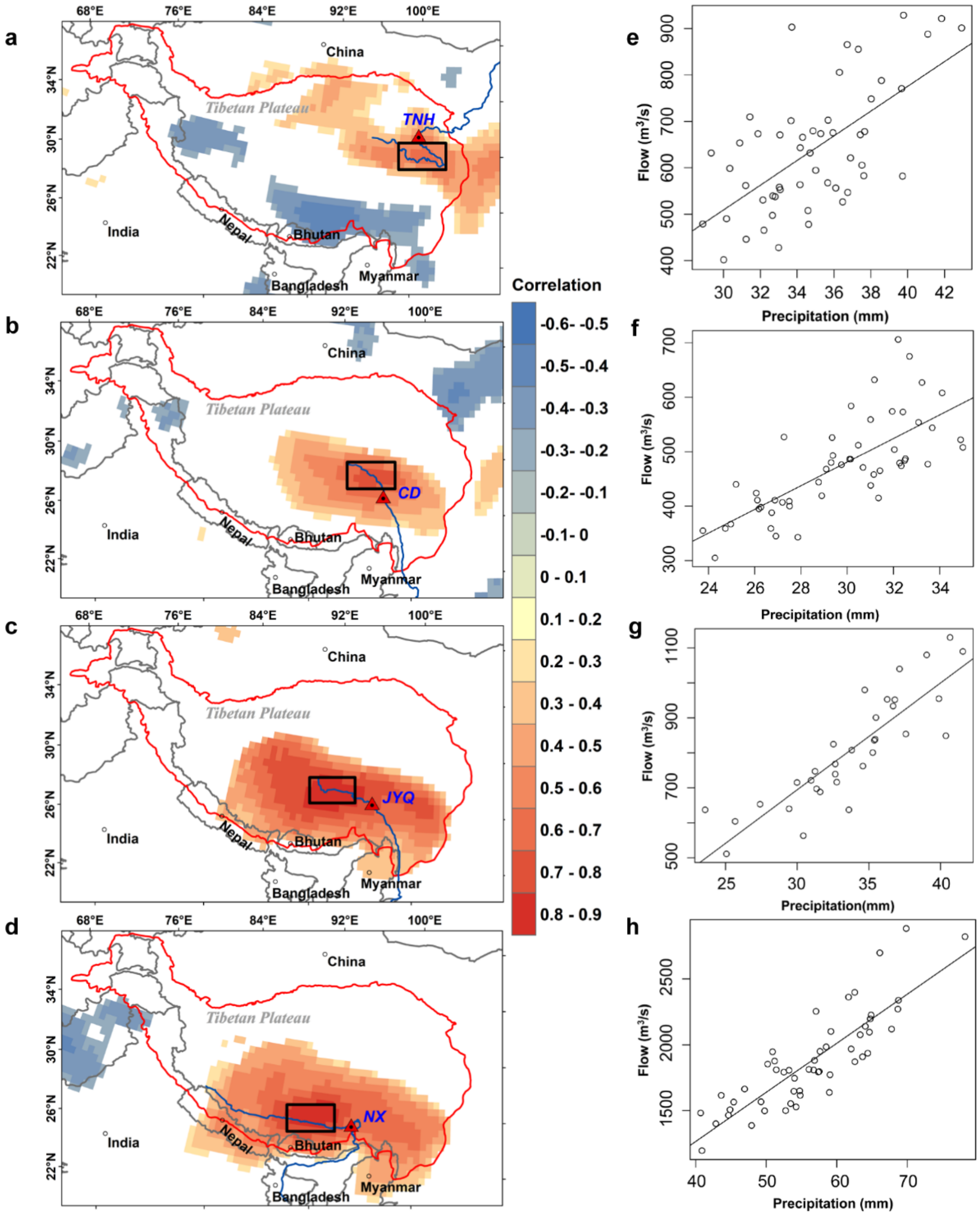

Supplementary Figure 6 Spatial correlation (left panel) and linear regression (right panel) between annual precipitation (CRU TS4.0) and reconstructed streamflow at the TNH (Yellow, a and e), CD (Lancang-Mekong, b and f), JYQ (Nu-Salween, c and g), and NX (Yarlung Zangbo-Brahmaputra, d and h) gauging stations. The black box (left panel) denotes areas used to average precipitation to perform linear regression (right panel) between streamflow at gauging stations and spatially averaged annual precipitation. Precipitation in the upper reaches of each river basin shows strong correlation with reconstructed streamflow at TNH (r=0.64, 1961−2012, p<0.05), CD (r=0.74, 1961−2012, p<0.05), JYQ (r=0.87, 1980−2012, p<0.05), and NX (r=0.85, 1961−2012, p<0.05).

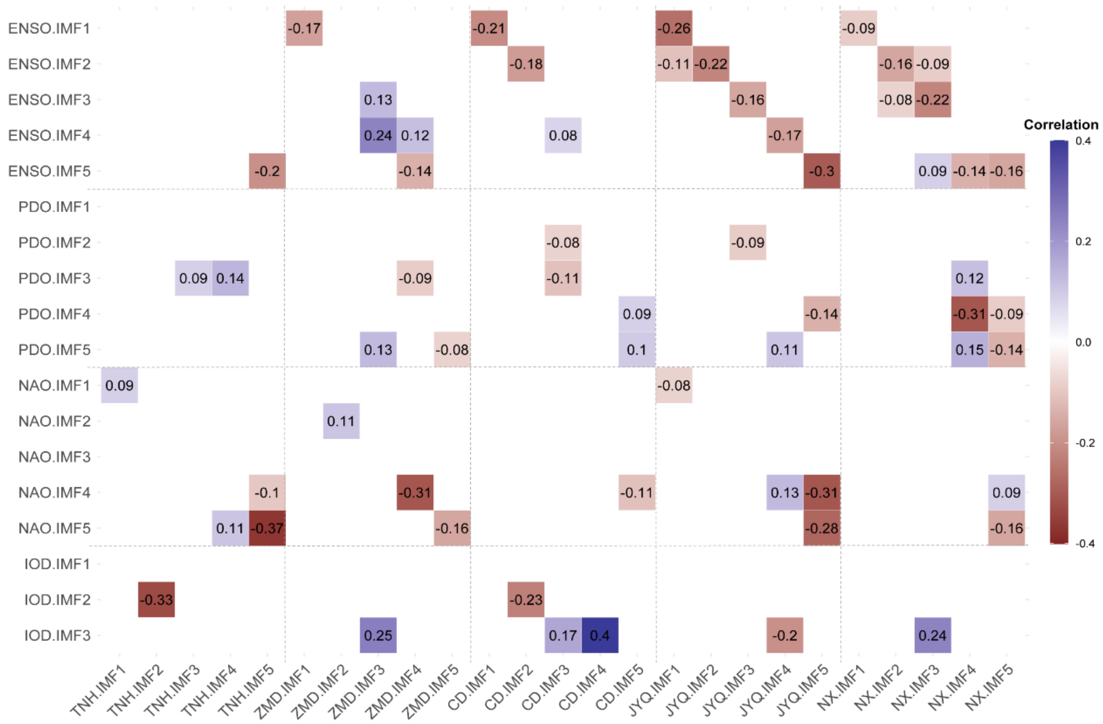

Supplementary Figure 7 Pearson correlation coefficients between each intrinsic mode function (IMF) component of reconstructed streamflow and large-scale climate patterns (i.e., ENSO, PDO, NAO, and IOD). Only significant correlation coefficients ($p < 0.05$) are shown. The IMF components with different frequencies for reconstructed streamflow and large-scale climate patterns were obtained using Ensemble Empirical Mode Decomposition (EEMD).

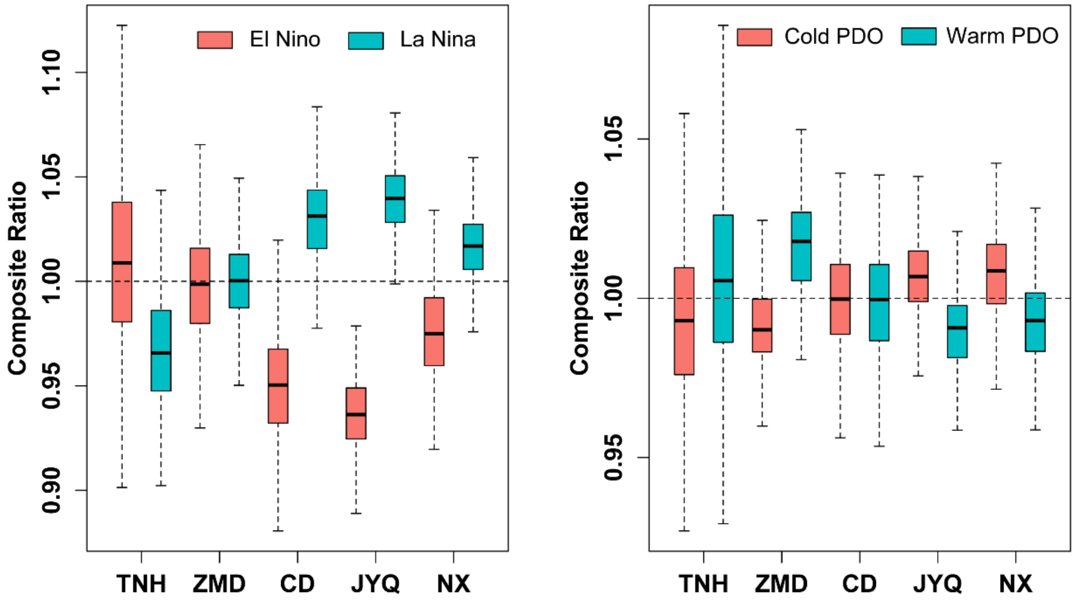

Supplementary Figure 8 Composite analysis of reconstructed streamflow at five streamflow gauging stations associated with different phases of ENSO (left panel) during 1301 − 2005 and PDO (right panel) during 1200 − 1996. A bootstrap resampling method was repeated 500 times to resample reconstructed streamflow values in certain extreme climate years to estimate the composite ratio.

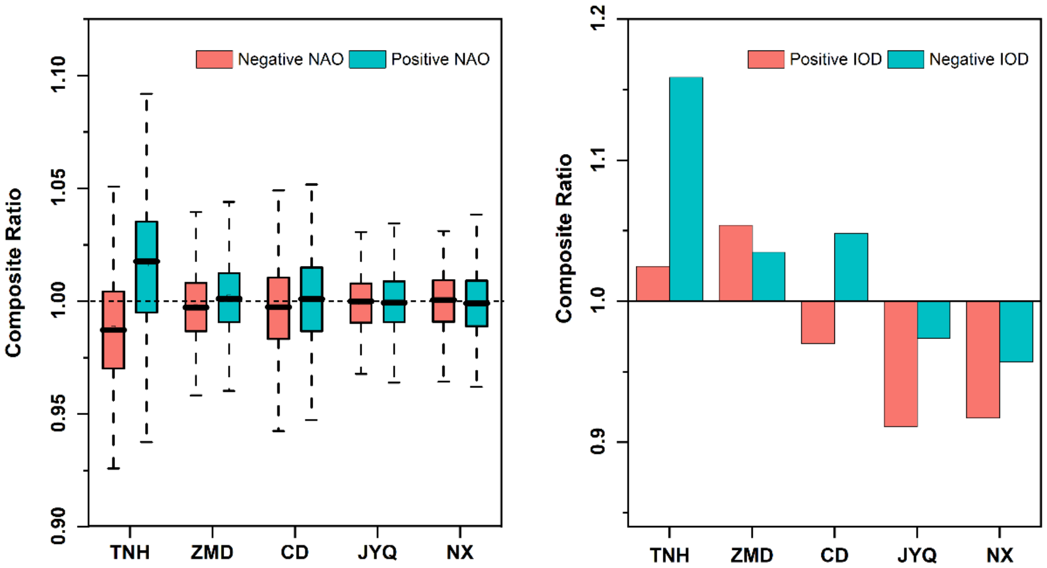

Supplementary Figure 9 Composite analysis of reconstructed streamflow at five streamflow gauging stations associated with different phases of NAO during 1400 – 2001 (left panel) and IOD during 1870−2012 (right panel). The bootstrap process was not used in the composite analysis between IOD and reconstructed streamflow during 1870 − 2012 because of the small sample size of extreme phases of IOD.

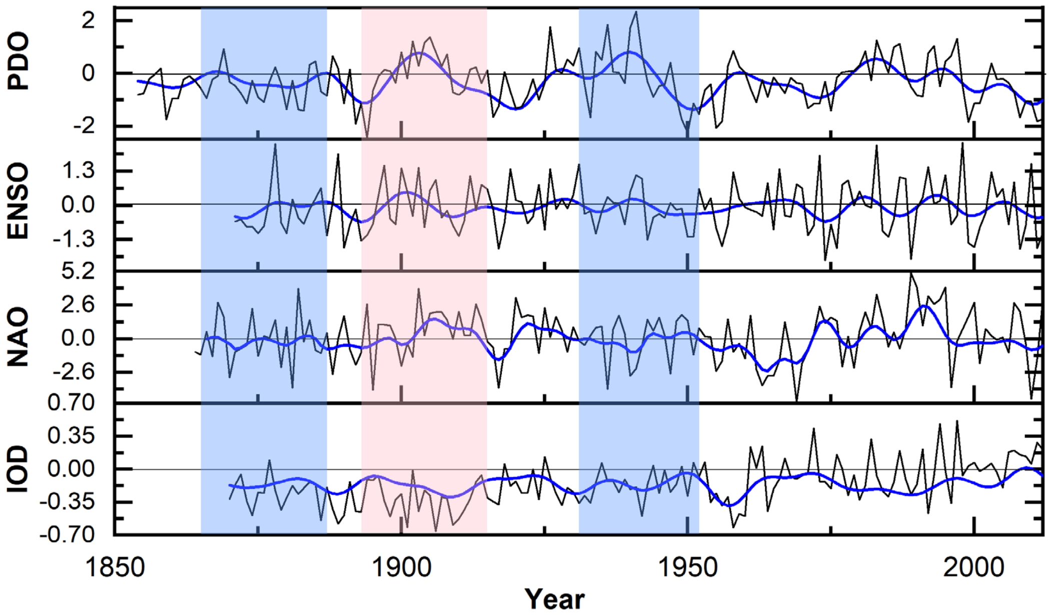

Supplementary Figure 10 Four observed large-scale climate patterns during 1850−2012 correspond to three contrasting wet and dry periods detected in the northern and southern SETP. Black lines denote observed data for ENSO, PDO, NAO, and IOD indices and blue lines represent their 10-year low pass series. Blue shaded areas represent the feature of contrasting streamflow variability, i.e., dry in the northern SETP (i.e., TNH, ZMD, and CD) but wet in the southern SETP (i.e., JYQ and NX) during 1865−1887 and 1931−1952. Red shaded areas denote the period of 1893−1915 experiencing a wet condition in the northern SETP (i.e., TNH, ZMD, and CD) but a dry condition in the southern SETP (i.e., JYQ and NX). PDO data are available here: https://www.ncei.noaa.gov/pub/data/cmb/ersst/v5/index/ersst.v5.pdo.dat, prior winter (November-January) ENSO data are available here: https://psl.noaa.gov/gcos_wgsp/Timeseries/Nino34/, winter (December-March) NAO data are available at this link: https://climatedataguide.ucar.edu/climate-data/hurrell-north-atlantic-oscillation-nao-index-station-based, and IOD data are available at this link: https://psl.noaa.gov/gcos_wgsp/Timeseries/DMI/.

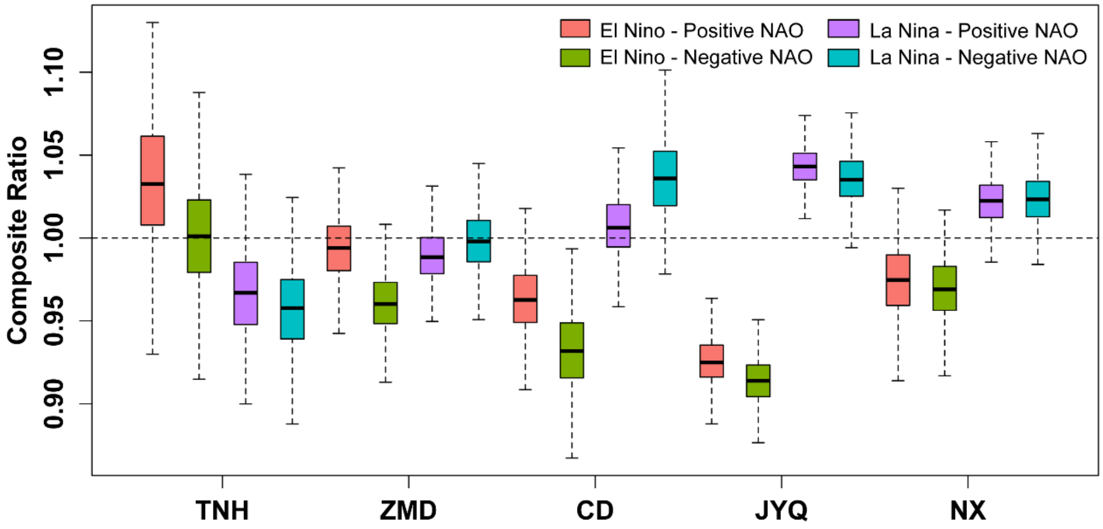

Supplementary Figure 11 Composite analysis of reconstructed streamflow in response to interactions between ENSO and NAO at five gauging stations. With different lengths of paleoclimate records, interactions between different climate indices on reconstructed streamflow were analyzed based on their overlapping periods.

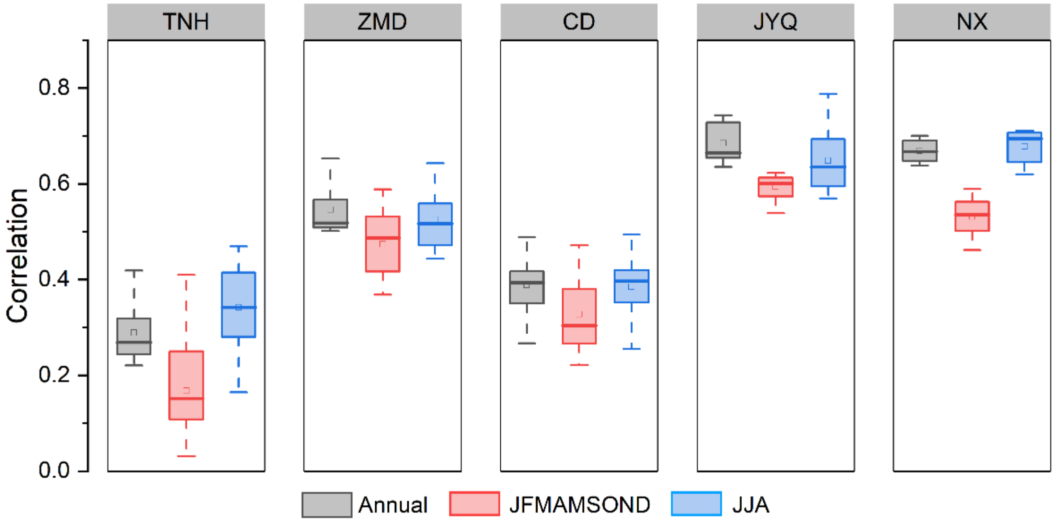

Supplementary Figure 12 Pearson correlation coefficients between PDSI predictors (June to August) and annual, June to August, and other nine-month (JFMAMSOND) streamflow at five gauging stations during 1961–2012. Correlation coefficients between PDSI and streamflow at three different timescales show little difference, indicating that June to August PDSI predictors can capture changes in annual streamflow well at the five gauging stations.

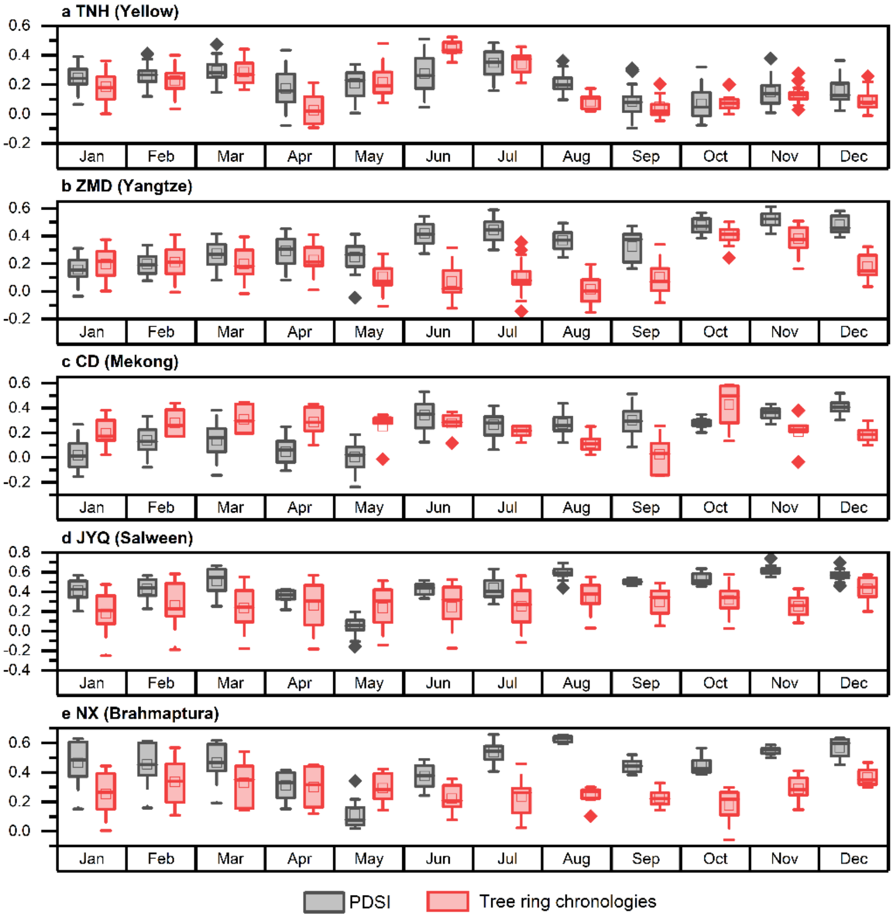

Supplementary Figure 13 Comparison of Pearson correlation coefficients for PDSI predictors (grey boxes) and tree-ring chronologies (red boxes) against monthly streamflow at five gauging stations. Tree-ring chronologies (totaling 80 sites, Fig.1a) located in or around the five river basins were downloaded from the International Tree-Ring Databank (https://www.ncdc.noaa.gov/data-access/paleoclimatology-data/datasets/tree-ring). The Auto-Regressive Standardization (ARSTAN) program was used to standardize raw annual tree-ring width data for detrending and removing some non-climatic factors in combination with a negative exponential curve. Appropriate tree-ring chronologies were selected as potential predictors based on the Pearson correlation coefficients ($p < 0.05$) estimated between annual mean flow at each streamflow gauge and tree-ring chronologies within a 700 km searching radius. Many tree-ring predictors end before the year 2012; hence, calculation of Pearson correlation coefficients was based on their overlapping periods.

Supplementary tables

Supplementary Table 1 Gauging stations selected for this study and detailed information on each gauge

River	Gauging Station	Longitude	Latitude	Data Availability	Annual Mean(m^3/s)
Yellow	Tangnaihai (TNH)	100.15	35.50	1961–2012	646
Yangtze	Zhimenda (ZMD)	97.24	33.01	1961–2012	417
Lancang-Mekong	Changdu (CD)	97.18	31.13	1961–2012	476
Nu-Salween	Jiayuqiao (JYQ)	96.24	30.87	1980–2012	807
Yarlung Zangbo-Brahmaputra	Nuxia (NX)	94.65	29.47	1961–2012	1896

Supplementary Table 2 Statistics of intrinsic mode function components at five streamflow gauging stations during 1200−2012 and four large-scale climate patterns.

Gauges / Climate Index	IMF components	IMF1	IMF2	IMF3	IMF4	IMF5
Tangnaihai	Main cycle	2−8	4−14	8−24	16−82	32−106
(TNH)	Correlation coefficient	0.52	0.38	0.4	0.34	0.37
Zhimenda	Main cycle	2−7	4−15	10−32	21−72	36−120
(ZMD)	Correlation coefficient	0.61	0.41	0.37	0.36	0.26
Changdu	Main cycle	2−7	4−14	14−25	15−48	24−116
(CD)	Correlation coefficient	0.64	0.42	0.34	0.28	0.27
Jiayuqiao	Main cycle	2−6	4−12	7−28	16−56	30−148
(JYQ)	Correlation coefficient	0.66	0.42	0.36	0.31	0.15
Nuxia	Main cycle	2−7	4−13	9−31	16−98	34−200
(NX)	Correlation coefficient	0.49	0.4	0.39	0.43	0.30
PDO	Main cycle	2−8	4−15	9−33	19−82	35−147
	Correlation coefficient	0.41	0.41	0.40	0.36	0.30
ENSO	Main cycle	2−7	3−14	7−20	12−19	22−94
	Correlation coefficient	0.68	0.50	0.39	0.25	0.15
NAO	Main cycle	2−6	4−12	7−26	26−93	55−222
	Correlation coefficient	0.79	0.45	0.23	0.14	0.10
IOD	Main cycle	2−7	4−12	7−39	/	/
	Correlation coefficient	0.62	0.32	0.31	/	/
